# Silence after narratives by patients in psychodynamic psychotherapy: a conversation analytic study

**DOI:** 10.3389/fpsyg.2024.1397523

**Published:** 2024-11-28

**Authors:** Carolina Fenner

**Affiliations:** Department of Pragmatics, Leibniz Institute for the German Language, Mannheim, Germany

**Keywords:** silence, turn-taking, psychodynamic psychotherapy, conversation analysis, narrative, therapeutic intervention, resistance

## Abstract

In psychotherapy, verbal communication is central to the therapeutic process. However, when patients remain silent, it can serve various functions, such as reflecting more deeply or hesitating to elaborate on a topic. This article uses conversation analysis to examine a specific context in which silence occurs: After a patient has concluded his/her narrative, both the therapist and the patient resist the turn allocation by the respective other, resulting in mutual silence. The results indicate that both therapists and patients collaboratively generate this silence. Therapists typically end the silence with an intervention, addressing an aspect of the topic and treating the pause as intra-topic silence. The study is based on approximately 29 h of video recordings of German-speaking outpatient psychodynamic psychotherapy sessions. This research highlights the importance of therapists recognizing the different forms of silence that may emerge during psychotherapy.

## 1 Introduction

In the context of psychodynamic psychotherapy,^1^ silence holds particular significance, as psychoanalytic psychotherapy is often referred to as the “talking cure” (Freud, 1910, p. 13): “the verbalisation of experience […] is a shared task for both patient and psychotherapist” (Bonacchi, 2021, p. 55). Consequently, the patient’s silence^2^ in psychodynamic psychotherapy was considered mainly in the earlier years “the most transparent and frequent form of resistance, […]. Generally, it means that the patient is either consciously or unconsciously unwilling to communicate his thoughts or feelings to the analyst” (Greenson, 2019, p. 61).

In more contemporary approaches, silence can still indicate resistance, but in specific contexts. More often, silence serves various purposes. For example, a patient may remain silent while preparing his/her next turn (König, 1995, p. 77). In psychodynamic psychotherapy, silence has also “been recognized as a meaningful contributor to the therapeutic relationship and valuable in assisting the patient to connect with his or her subjective experience at unconscious levels” (Knol et al., 2020).^3^ Furthermore, silence is not necessarily a solitary occurrence; extended silence can be seen as a joint discourse activity, during which both participants’ internal mental processes continue to develop (Thomä and Kächele, 2006, p. 312).

Although silence is an important element of psychodynamic psychotherapy, there is a lack of conversation analytic studies that examine the context in which silence occurs, what happens during silence (concerning gazes and embodied actions), and how silence in psychodynamic psychotherapy is treated by the participants.

When analyzing the data, I found two major contexts in which silence occurs most often when both participants resist turn allocation by their conversation partner: (a) the patient tries to allocate the turn to the therapist, who does not take it but remains silent; (b) the therapist tries to allocate the turn to the patient, who does not take it but remains silent. Due to the limited scope of this article, I will focus on type (b) and only include instances in which therapists resolve the silence. Hence, this article addresses the following questions: How do patients indicate they already finished their turn, and how do they try to allocate the turn? How do therapists show that they expect the patient to say more? How do therapists resolve silence?

## 2 Theoretical framework

### 2.1 Silence in conversation analysis (CA)

Generally, conversational silence is defined by Bilmes (1994, p. 79) as “the absence of talk (or of particular kinds of talk) where talk might relevantly occur” (see also Schegloff, 1968, 2007, pp. 19–20; Schegloff and Sacks, 1973, p. 294).^4^ Such silence is to distinguish from “freien Gesprächspausen” (‘free pauses in conversation’), as Bergmann (1982, p. 153) calls silence for which none of the participants in a conversation can be held responsible, as no next speaker has been implicitly or explicitly selected. There are difficulties in the assessment of silence concerning embodied behavior that takes place during it, but even more so with vocal productions such as sighs and loud exhalations. The literature is unclear whether silence includes such sounds or whether a pause in speech is then not categorized as silence. Moreover, it is difficult to analyze conversationally what functions such sounds have. According to the turn-taking system, as described by Sacks et al. (1974), a speaker—somewhat simplified—either chooses the next person to take the turn or the next person chooses him−/herself. A transition from one turn to the next usually occurs at a so-called transition-relevance place (TRP). “Transitions […] with no gap and no overlap are common” (Sacks et al., 1974, p. 708). Mostly, turn transitions between first and second actions occur with a gap of 100–300 ms (Levinson and Torreira, 2015, p. 11), whereas “gaps longer than the norm (>300 ms) decrease the likelihood of an unqualified acceptance, and increase the likelihood that a response […] will have a dispreferred turn format” (Levinson and Torreira, 2015, p. 3; see also Levinson, 1983, p. 299).

In CA literature, there is a distinction between different types of silences called pause, gap, and lapse. The classification rests upon the analysis of the placement of silence within the turn-taking system (Hoey, 2020, pp. 8–9): Pauses are intra-turn silences (Sacks et al., 1974, p. 715) “that occur before a turn has come to possible completion” (Hoey, 2020, p. 9), i.e., they ‘belong’ to a speaker. “Gaps are silences that occur after possible turn completion and before the beginning of a next turn” (Hoey, 2020, p. 10); that is, they ‘belong’ to the selected next speaker, who usually tries to minimize the occurrence and duration (Hoey, 2020, pp. 9–10). The current speaker may decide to continue after such a gap, which is then transformed into a pause (Hoey, 2020, p. 10; Sacks et al., 1974, p. 715). In turn, a lapse may occur when the current speaker has stopped, and no other speaker has been selected, thus the conversation discontinues. A lapse appears only at sequence endings when no specific action is projected to come next, and all participants can self-select (Hoey, 2020, pp. 11, 17).

**Figure 1:**
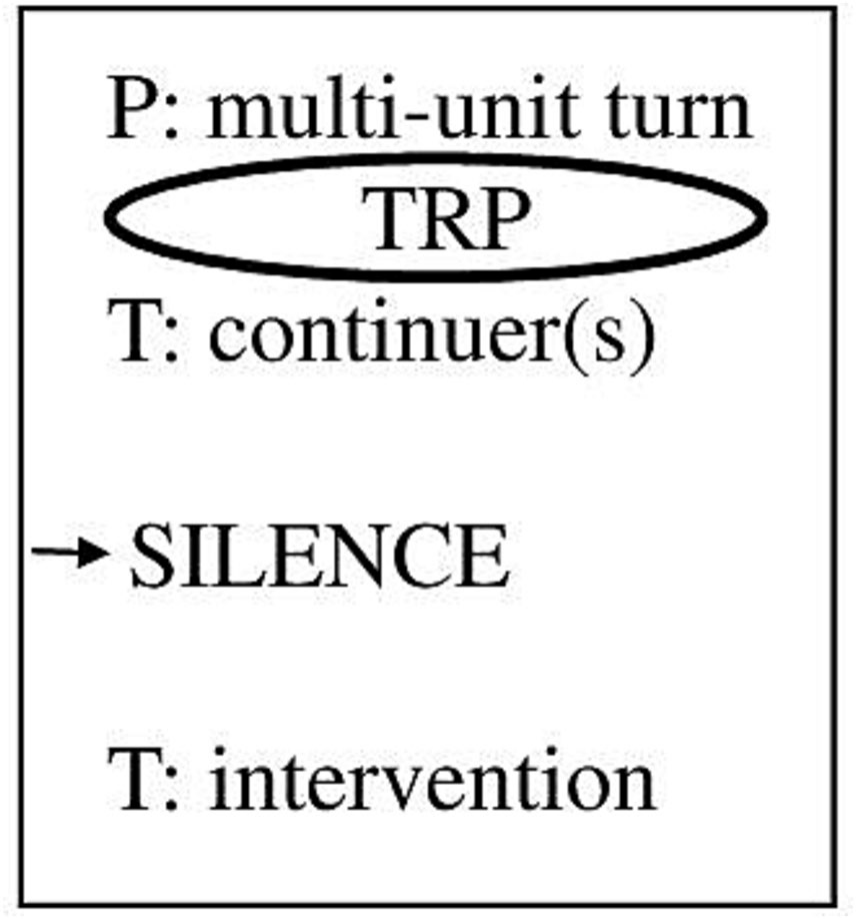
Sequential focus of study.

To answer the above-stated research questions, I will focus on silences after a therapeutic verbal continuer after a TRP in dyadic psychotherapeutic interactions, which occur at the end of a narrative^5^ by the patient, that is, a multi-unit turn. With the continuer, the therapist displays that s/he expects the patient to continue. In addition, I will only deal with cases in which the silence is ended by a therapeutic intervention. Figure 1 illustrates the type of silence I will focus on.

In my data, I observed more frequently that patients respond to the therapists’ continuers by telling more or that the therapists themselves almost immediately after their continuer produce some intervention. However, this is precisely why the instances are interesting in which patients do not comply with the therapeutic request to continue, and therapists first wait instead of directly taking the turn.

### 2.2 Silence in psychotherapy

In psychotherapy, remaining silent for a longer period is much more common than in mundane talk-in-interaction (Chatwin et al., 2018, p. 12; Levitt, 2001, p. 221)—regardless of the specific type of therapy. Therefore, the standard maximum silence of approximately 1 s in ordinary talk (cf. Jefferson, 1989) does not apply (Berger, 2011, p. 304). Usually, conversation analysts examine silence in psychotherapy longer than 3 s (cf. Buchholz, 2018, p. 97; Knol et al., 2020) because shorter silences are regarded as dysfluencies (Levitt and Morrill, 2021, p. 234), so I adopted this criterion. There are countless essays and studies on silence in psychotherapy, but most of them are quantitative or, if qualitative, based on interviews. Interactional or conversation analytical studies on silence in psychodynamic psychotherapy are rare. In the following section, I will present the conversation analytic studies that are relevant to the subsequent analysis.

Muntigl and Zabala (2008) analyze two cases of couples’ therapy and show that therapists can use silence as an expansion technique to get the client to say more, among other expansion techniques such as continuers and prompts.

Berger (2011) explores lengthy silences in a psychotherapy session and concludes that they “can be present in conversation as an unproblematic and regularly occurring feature” (Berger, 2011, p. 304). Rather, it depends strongly on the respective context and how silences are treated.

Similarly, Pawelczyk (2011), who analyzes a 65-h corpus of psychotherapeutic interactions from one psychotherapist with 25 patients, claims that silences in psychotherapeutic discourse can index therapeutically relevant material to be disclosed by the patient (Pawelczyk, 2011, p. 181).

Chatwin et al. (2018) explore aspects of silence in telephone-delivered cognitive behavioral therapy and call gaps, which turn into pauses, ‘same-party prompt’, and ‘same-party repair’—depending on what happens after the silence. *Same-party prompts* are “associated with a degree of ambiguity in the nature of the [preceding] turn” (Chatwin et al., 2018, p. 8). In those cases, there seems to be some kind of discrepancy between what the first speaker is trying to project and what the other interlocutor takes this to mean. In turn, *same-party repairs* are “associated with the misinterpretation of TRP indicators” (Chatwin et al., 2018, p. 11), with more evident repair and remedial actions after the silence by the speaker who owned the turn before the silence.

Knol et al. (2020) analyze silence in psychodynamic psychotherapy that occurs after a therapeutic continuer that follows a patient’s turn. Their study shows “that silence (1) can retroactively become part of a topic closure sequence, (2) can become shaped as an intra-topic silence, and (3) can be explicitly characterized as an activity in itself that is relevant for the therapy in process” (Knol et al., 2020). They state that silences usually occur cumulatively and that patients tend to talk with a more emotional stance after silence. Only in case (3) silence is treated as disruptive to the ongoing conversation.

In contrast, Voutilainen and Koivisto (2022) examine 18 sequences from six sessions of two dyads from psychodynamic psychotherapies, in which the patients express a negative experience—typically a complaint—but the therapists do not respond, thus the patients back down from their earlier affective stance, which is responded by the therapists with an account by which they show delayed empathy. Voutilainen and Koivisto (2022, p. 263) call the timing of the therapeutic response an interactional means “through which the therapists attune to the clients’ emotional conflicts.”

All studies show that both conversational parties in psychotherapy can use silence interactionally in different ways, for example, by therapists as a practice to elicit expansions and by patients as resistance to continuing their turn or responding.

My article will tie in with Knol et al.’s (2020) study but focus more on what happens in the sequences where the therapists continue to pursue the topic after the silence. Which practices do therapists use? What interpretation of the patients’ actions do the therapeutic interventions imply, and what therapeutic purpose does this serve?

## 3 Materials and methods

### 3.1 Data

This study is part of the DFG-funded, interdisciplinary project *Linguistic Manifestations of Resistance in Psychodynamic Psychotherapy* run by the Leibniz Institute for the German Language (IDS) and Department for General Internal Medicine and Psychosomatics Heidelberg. Video recordings of outpatient therapy sessions were conducted at the Heidelberg Institute for Psychotherapy (HIP), a psychodynamic psychotherapy training institute of Heidelberg University Hospital. The HIP is a state-approved training institute for psychological psychotherapists. The outpatient therapies are continuously videographed for quality assurance, supervision, and process research. For the study at hand, a sample of *N* = 32 videotaped psychotherapy sessions (randomly selected sessions number 5, 15, and/or 30) from 28 different patients was drawn from the available video data. Written consent from patients and therapists for using the data for psychotherapy research was available, and the Ethics Committee of the Medical Faculty of the University of Heidelberg approved the use of the data for this purpose (AZ: S-2020/2020).

### 3.2 Method

I searched in the whole sample for silence with a duration of at least 3.0 s, which occurs after the therapist utters a verbal continuer in the *transition relevance space* after a TRP at the end of a patient’s multi-unit turn (a narrative). I only examine silence, which is ended by the therapist’s turn.^6^ I excluded all cases in which the patients and/or the therapists make any verbal sounds during the silence, for example, breathing loudly, coughing, sighing, as well as four cases in which therapists changed the topic after the silence.^7^

In total, I identified 21 cases within 11 different sessions from 10 different patients with nine different therapists. All cases were transcribed according to GAT 2 conventions (see Selting et al., 2011; a legend of the symbols can be found in Appendix 1). I also prepared multimodal transcripts according to Mondada (2018; a legend of the symbols is given in Appendix 2) because “the participants’ embodiment – their gaze, body posture, bodily activities, etc. – has a crucial role in understanding and interpreting silences” (Vatanen, 2021, p. 326) and the respective turn-taking, as will be shown in the analysis section. In my collection, the silence duration varies from 3.0 s to 13.7 s. The average duration of the 21 instances of silence is 5.5 s.

## 4 Results

In my collection, all patients show with their silence that they had finished their turn and do not intend to add anything regarding the issue. However, instead of displaying unwillingness, they often indicate a need for assistance by the therapists. Therapists treat the silence (and the previous turn by the patient) differently. However, in all cases, they focus on a specific aspect and display that the topic has not yet been treated sufficiently. In most cases (*N* = 11), they ask a question, thus making an answer from the patient conditionally relevant. Hence, they indicate that they treat the previous silence as misaligning and expect the patient to elaborate more on the topic. Nevertheless, asking questions might also provide help. Furthermore, therapists often deliver an interpretation (*N* = 6), thereby opening up a new perspective to the patient. Therapists rarely use two other practices: They can use challenges (*N* = 2) to give the patient new input regarding the content and make a reaction relevant. Moreover, therapists can apply formulations (*N* = 2) to focus on and deepen a specific aspect of the patient’s narrative. With the last two interventions, they can also help the patient by showing empathy or supporting them.

### 4.1 Therapeutic question

Therapists usually (*N* = 11) break the silence with a question, thus making an answer from the patient clearly relevant and delving deeper into a specific aspect of the current topic. Simultaneously, the question might help the patient, for example, to elaborate. I will explain this in more detail below.

Before the beginning of excerpt (1), P^8^ says she misses having something exclusively on her own because she is with her husband all day, and she feels that she has to justify every single move. She thinks it would help if she could get some distance and hopes it would give her some peace and quiet. As an example of that feeling, she talks about a flower shop nearby where she always stops at the window. She experiences the decoration in the window as something calming, which puts her in ‘a different state of mind,’ as she calls it, and admits that it is difficult to describe:

(1) Pat24_T15 min. 14.41^9^^,^^10^

Initial position: P has both hands in her lap. T has a writing board on her lap, which she holds with her left hand. In her right hand, which rests on the board, she holds a pen.
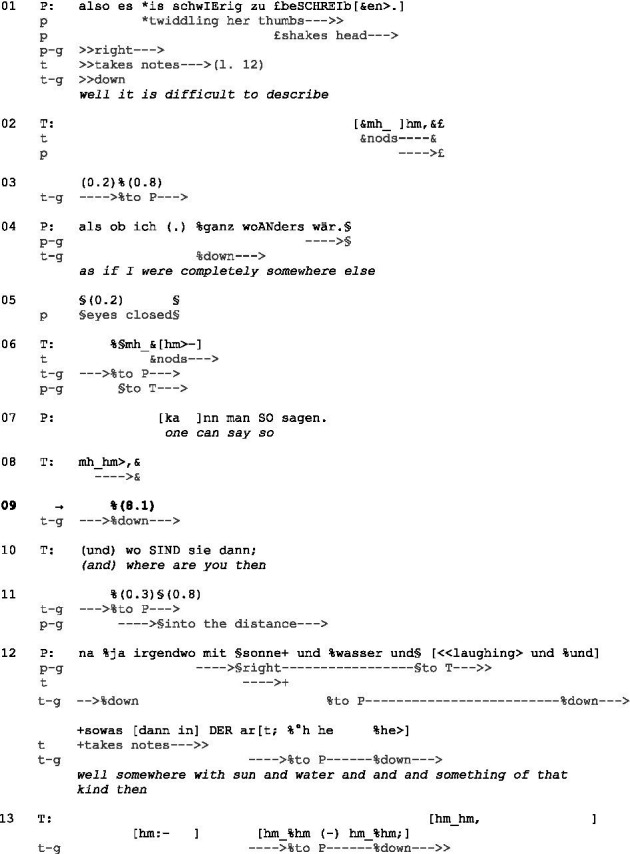


While P is talking, T takes notes and shows that she is listening to P regardless by producing continuers. A possible TRP occurs after each TCU, which is characterized by falling intonation and syntactic completeness (lines 1, 4, 7). However, P looks to the right and not to T, which can be a sign that she is still trying to formulate this ‘different state of mind.’ T utters several continuers, thus showing she expects P to say more and does not intend to take over the turn. P closes her turn by stating ‘one can say so’ (line 7) with falling intonation and looking at T, indicating that she allocates the turn to her (cf. Kendon, 1967, pp. 35–36). However, T utters a continuer (line 8) at this TRP: She “embodies the understanding that extended talk by another is going on by declining to produce a fuller turn in that position” (Schegloff, 1982, p. 82). Then, T continues writing and looks back at her notes during the following silence of about 8 s. P, meanwhile, looks straight at T and continues twiddling her thumbs as she has done since the beginning of the excerpt. This could be a sign of uncertainty or nervousness.

Of course, one could assume that the silence arises because of T’s preoccupation with taking notes. However, the context contradicts this since T also listens and takes notes simultaneously or speaks and takes notes simultaneously throughout the therapy session. Furthermore, P is not always silent while T is writing but sometimes continues talking, irrespective of this.

In any case, P shows no intention of continuing her turn. Instead, T interrupts the silence by asking a specific question: ‘(and) where are you then’ (line 10) and gazes at P. By asking this question, which aims at specifying P’s previous statement, she indirectly indicates that P’s statement before about ‘somewhere else’ (line 4) was too vague and not defined by asserting properties (see also Spranz-Fogasy et al., 2020). Possibly, the previous continuer was already aiming at P to explain this in more detail. However, the continuer and the silence did not project a specific type of action. At the same time, by asking this question, T helps P to continue with her elaboration and to share what is on her mind. P then provides an answer while looking partly into the distance. Thereby, she shows visibly that she is ‘somewhere else.’ T acknowledges her answer with multiple “hm_hm” (line 13).

By refraining from taking the right to speak, T made clear that she expected P to elaborate autonomously (Deppermann, 2009, p. 155). In doing so, she ascribes to P the competence in the sense that ‘you can do it on your own; you do not need my help.’ Since P does not fulfill this requirement—possibly seeking help—T indicates more explicitly which subsequent action she expects by asking a question. Thereupon, P meets this request and provides an answer (line 12).

Example (1) shows that P tries to allocate the turn to T while T denies it but indicates that she expects P to expand her turn. P’s silence might be interpreted as ‘I have nothing more to say’ or ‘I cannot do this on my own. I need your help.’ When T’s continuers no longer have any effect and P shows no willingness or ability to continue her turn, T uses another intervention and asks a specifying question. Thereby, she makes an answer from P clearly relevant, who fulfills this expectation but remains rather vague.

### 4.2 Therapeutic interpretation

Also comparatively often, therapists express interpretations after silence (*N* = 6). With these interventions, they stay on topic and deliver their view since therapeutic interpretations include the therapists’ thoughts, associations, and feelings (Stukenbrock et al., 2021, p. 77). Interpretations usually make relevant an acceptance or a rejection on the part of the patient. In case of an acceptance, an elaboration is required (cf. Peräkylä, 2008). The following example (2) illustrates silence with a therapeutic interpretation afterward. Before the extract, P complains about her boyfriend. T addresses an earlier interpretation of herself and repeats that there might be a(n) (unconscious) pattern match between the relationship of P with her partner and her relationship with her mother. She asks whether P can follow this. P cautiously agrees and thereby accepts the interpretation.

(2) Pat14_T30 min. 24.51

Initial position: P has crossed her legs. Her arms rest in her lap, and she gazes at T, who has also crossed her legs. Her left arm rests on her right leg. Her right arm rests on the armrest, and her chin leans on her right hand. She gazes at P.
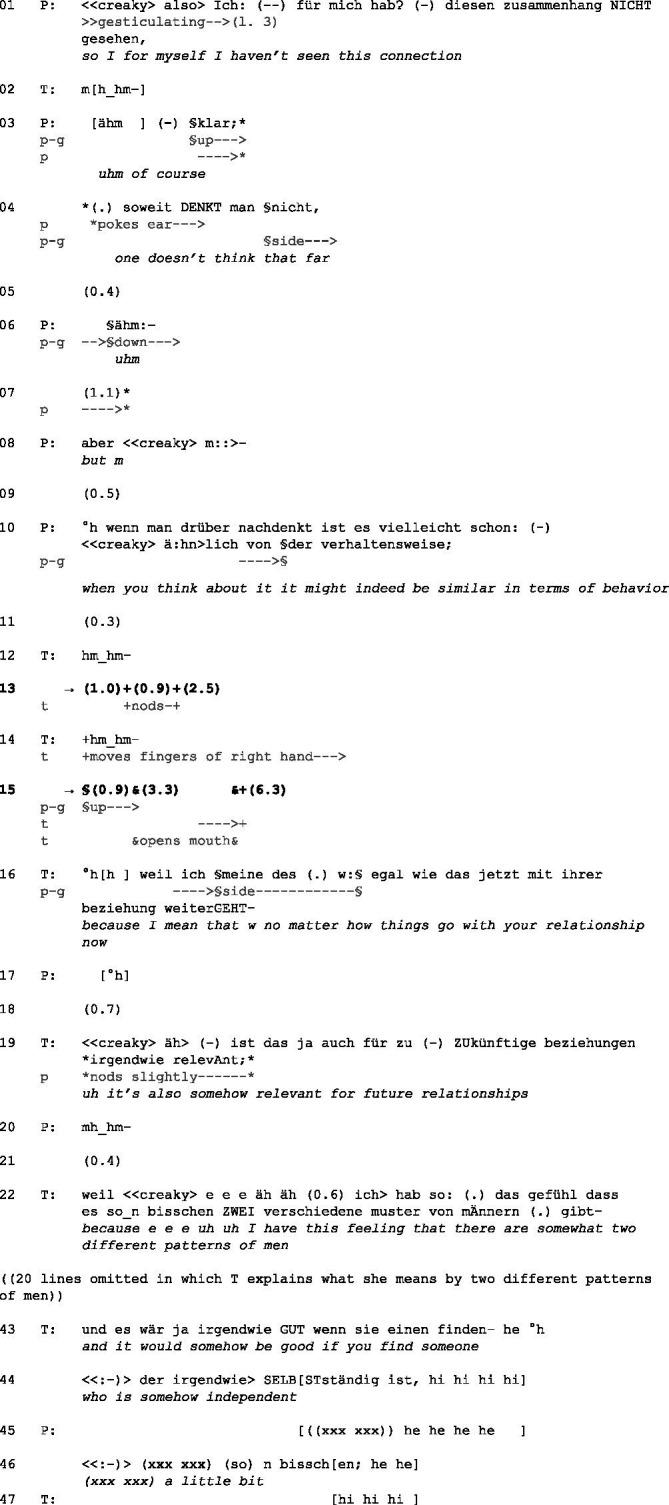


P stays rather vague by using the impersonal “man” (‘you’/‘one,’ lines 4, 10) and the adverb ‘maybe’ (line 10). Before the end of her turn, P shifts her gaze to T (cf. Stivers and Rossano, 2010). She completes her turn syntactically and shows with falling intonation that she allocates the turn to T. T reacts with a verbal and an embodied continuer—“hm_hm” (line 12) and nodding (line 13)—thus allocating the turn to P instead. After a pause, she utters another continuer (line 14). Thereby, she reinforces her turn allocation to P, who remains silent. By looking up, P displays that she is no longer available to be the next speaker. She might imitate a thinking pose (cf. Heller, 2021, p. 8), thus pretending she is occupied. Since her previous statement was vague, her silence may indicate that she does not commit herself and, therefore, has nothing more to say.

T starts moving her fingers and opens her mouth for more than 3 s (line 15) but then closes it without saying anything. No inhalation or attempt to speak can be heard, so it remains unclear whether T indicates that she is about to take the turn. However, P does probably not even notice this since she is looking up. This is followed by another 6 s of silence (line 15), in which neither T nor P shows any attempt to take the turn. Finally, T breaks the silence by uttering an extended multi-unit turn to expand her interpretation. She addresses the consequences of this pattern match for future relationships. She explains to P that she has had relationships (not always in a romantic sense) with two different types of men. In future relationships, however, it may be a matter of having them with a third type of man, independent men (lines 16–44). She accompanies her interpretation with humor and laughter (line 43). P affirms, for example, by nodding (line 19), joining T’s laughter (line 45), and commenting (line 46). The moment in which P joins T’s laughter might be considered an intersubjective moment in the therapy process, even a moment of meeting (Stern, 2004), in which mutuality is achieved through practices of “doing-we” (Buchholz, 2022). After the extract, T finishes the expansion of her interpretation, and another silence ensues. P finally accepts the interpretation cautiously again but does not elaborate on it. Instead, she shifts the topic toward her concerns about what others might think if she breaks up with her boyfriend [cf. extract (3)].

In this case, P clearly allocates the turn to T through interactional devices such as gaze, syntactical closure, and falling intonation at the end of her turn. She then stays silent and even presents herself as unavailable as a speaker. Her silence might be interpreted as ‘I have nothing more to say’—and thus could be considered resistant. T utters continuers, thus allocating the turn to P. Since T started her interpretation before and P accepted it—although very cautiously—T is waiting for P to elaborate. However, T finally breaks the silence with an extension of her interpretation. By expanding her interpretation, T “propose[s] something that the patient might not have been aware of” (Peräkylä, 2011, p. 289). Interestingly, T does not address P’s difficulties in accepting her interpretation but expands and explains it.

### 4.3 Challenging intervention

Therapists can also interrupt the silence by expressing a challenging intervention afterward, thus giving the patient new input regarding the content and making a reaction relevant. Though therapists use a challenge, they can—at least partly—show empathy or understanding at the same time. However, this happens rarely (*N* = 2).

In example (3), P talks about the possible end of her relationship with her partner and admits that she wants to please everyone but herself.

(3) Pat14_T30 min. 27.23^11^

Initial position: P has crossed her legs, her right hand is under her left leg, and her left arm rests on the armrest. T mirrors her position beside her right arm that does not rest on her armrest. Instead, her chin rests on her right hand. P looks down while T gazes at P.
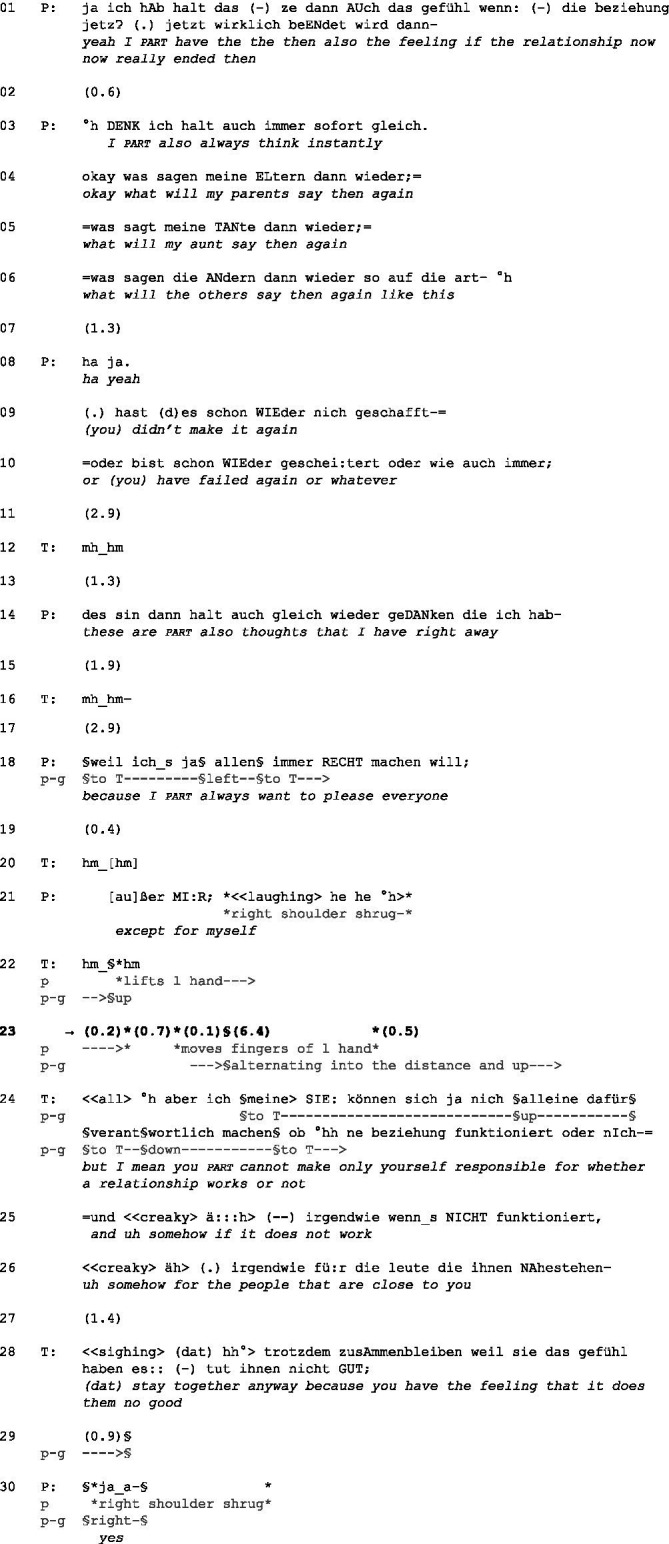


P talks about potential difficulties that would result from ending her current relationship with her partner (line 1). She verbalizes the problem that she is always afraid of what others might say, for example, her parents and her aunt (lines 3–5). Then, she quotes what she expects to hear from others (cf. lines 8–10) and ends her turn with ‘or whatever’ (line 10; cf. Leuschner, 2000, p. 352) and falling intonation. However, T does not take the turn but utters a continuer (line 12) after remaining silent for almost 3 s (line 11). A continuer does not make a particular action relevant but indicates that T still assigns the right to speak to P. Deppermann (2009, p. 155) and refers in this context to an allocation of the duty to speak (in German “Redepflicht”). After another short silence (line 13), P recompletes her turn by adding, ‘these are also thoughts that I have right away’ (line 14). The intonation does not fall in the end, but through syntactic closure and silence afterward, it becomes clear that another TRP emerges, and P tries to hand over the turn to T. The use of the particle “halt” (line 14) also contributes to the end of her utterance being a TRP because “*halt*-utterances often have a […] sequence and topic closing function” (Betz, 2015, p. 115).

Silence emerges (line 15), which might be an invitation to T to comment on what P has just said. However, T utters another continuer (line 16), not showing any intention to say more. Consequently, P expands her turn and summarizes that a part of her always wants to please everyone but herself (lines 18–21). This is the first time in this extract she moves away from factual matters and talks about her feelings instead. It represents an important and serious insight within the context of psychotherapy. By using the modal particle “ja” (line 18), she displays that her statement is not only part of the common ground but also evident and unquestionable (Reineke, 2018, p. 194). Nevertheless, P laughs afterward, thereby framing her talk as something delicate (Haakana, 2010, p. 1510) and possibly displaying discomfort by laughing. Simultaneously, she shrugs her right shoulder and, afterward, lifts her left hand like a hand shrug and looks up. In this way, she might display resignation and embodied not-knowing (Debras, 2017). She does not know how to change, so she might have difficulties dealing with this issue on her own. Additionally, she marks the end of her turn with falling intonation. T reacts again with a continuer, thus still assigning the right to speak to P. However, P does not talk further but keeps silent. Instead, she alternates between looking up and into the distance, showing that she is unavailable as the next speaker (cf. Goodwin, 1980). In addition, she moves the fingers of her left hand slightly, possibly displaying discomfort. However, after almost 8 s, T breaks the silence with an implicit rejection in the form of an account that presents a counterargument (cf., among others, Amar et al., 2022): P cannot only make herself responsible for whether the relationship works out (cf. line 24–26). T challenges the part of P, which criticizes her and simultaneously endorses the other part of the conflict—the one that recognizes her tendency to please everyone but herself. Hence, T presents herself as an ally of that part of P. However, P only responds minimally (‘yes,’ line 30) with an annoyed voice.

Extract (3) shows that P tries to allocate the turn to T, who does not take it but instead uses continuers, signaling that she expects P to continue speaking. P’s subsequent silence might be interpreted as ‘I do not know what to do’ and ‘I cannot get any further on my own.’ Hence, P’s silence might also serve as an implicit request for T to step in. Finally, T uses a challenging intervention to both confront the neurotic part and endorsing the reflective side of P. In essence, both interlocutors perceive the other’s silence as a form of disalignment although the silence is co-constructed interactively by both participants.

### 4.4 Therapeutic formulation

Just as rarely as challenging interventions, therapists use formulations to break the silence and to “show understanding of the previous speaker’s [the patient’s] turn by proposing a version of it” (Weiste and Peräkylä, 2013, p. 300; see also Heritage and Watson, 1979). Formulations usually make a (dis-)confirmation by the patient conditionally relevant. Example (4) shows a case where the therapist interrupts the silence with a rephrasing formulation (cf. Weiste and Peräkylä, 2013, pp. 306–309).

Before the extract, P started telling how he and his partner had gone to a bike store to buy a new bike.

(4) Pat20_T30 min. 40.47

Initial position: P sits sideways to T with crossed legs. His hands are folded together in front of his chest, and he gazes to the side (away from T). T has also crossed her legs. Her left arm rests on her stomach. Her right arm rests on her left hand, and her right fingers touch her mouth. She gazes down.
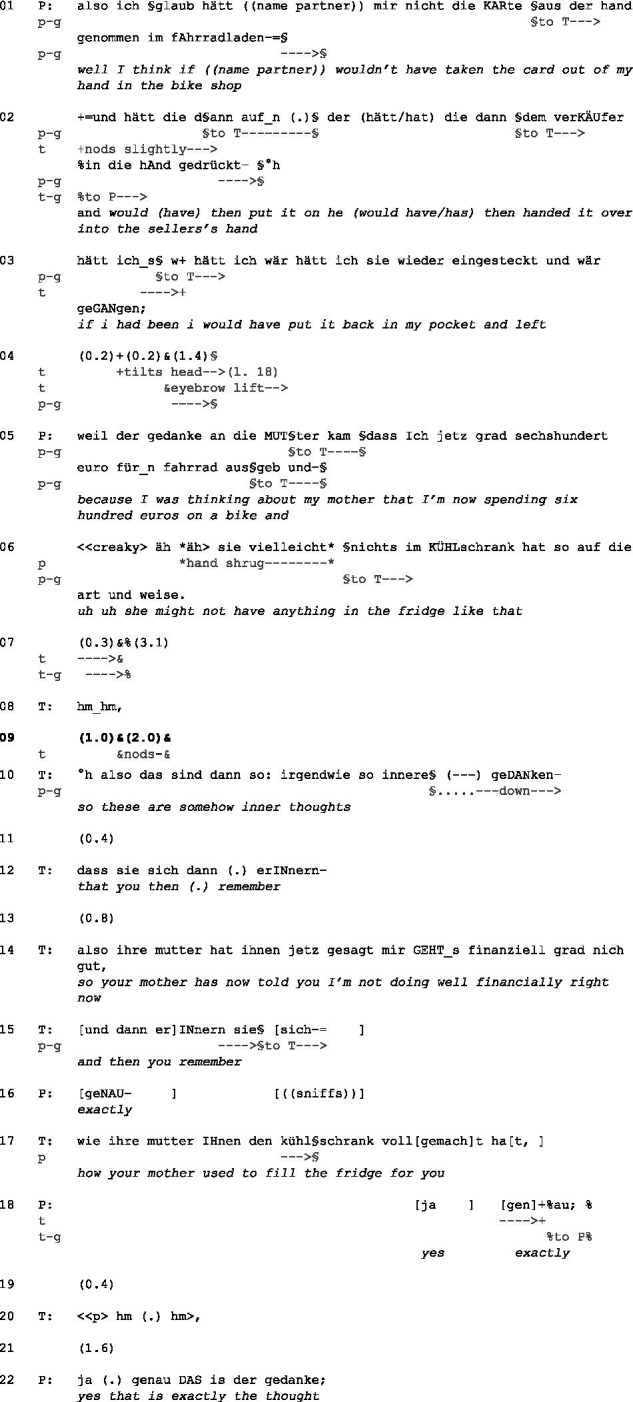


P says that he was about to leave the store without buying a bike if his partner had not taken his card to pay. The syntactic completeness, the slightly falling intonation, and the gaze to T (line 3) lead to a first TRP. However, T raises her eyebrows questioningly and tilts her head (line 4). Thereupon, P explains what was going on inside him (lines 5–6). Another TRP emerges, which is again indicated by syntactic completeness, falling intonation, and P’s gaze on T. In addition, he shrugs his hands, possibly displaying despair but also unchangeability (cf. Debras, 2017). In the resulting pause (line 7), P continues looking at T in the apparent expectation of a reaction from T. However, T stops the mutual eye contact by looking down and utters a continuer. T indicates that—also due to her previously questioning posture, which she has not completely resolved—she expects a further explanation from P. However, P remains silent, and after 1 s, T starts nodding (line 9), which can be understood as a non-verbal continuer. After a total of 3 s of silence, T comes in with a rephrasing formulation: She proposes her own version of P’s story in generic terms, focusing more on the subjective meaning of P’s experience (cf. Weiste and Peräkylä, 2013, p. 306). Overall, she articulates this with rising intonation in a rather questioning manner. Formally, she shifts the focus toward P’s feelings and makes a reaction from him relevant. In the case of a rephrasing formulation, this is even an extended (dis-)agreement (Weiste and Peräkylä, 2013, p. 306). However, in terms of content, she offers help with this formulation, showing that she interpreted P’s silence as a request for help.

Example (4) shows how P tries to allocate the turn to T, while T indicates that she expects P to expand his turn. However, P shows no willingness to continue his turn. His silence might be interpreted as despair but also as seeing no possibility for change. T then uses a rephrasing formulation, implicitly addressing the disalignment of the previous silence, to offer her reading of a key aspect (Weiste and Peräkylä, 2013, p. 306): P’s feelings. Thereby, she makes a response from P clearly relevant.

### 4.5 Summary of results

The examined type of silence that I have studied in this article occurs relatively rarely—only 21 times in 10 therapy sessions. Hence, in 22 psychodynamic psychotherapy sessions in my sample, no silence with a length of at least 3.0 s occurred after a therapeutic continuer following a narrative by the patient that was ended by the therapist.

Patients allocate their turn to the therapists by using several interactional devices, such as syntactical and prosodic means and gaze. With their embodied behavior, they indicate that they have nothing more to say—although they stayed rather vague before—or that they feel desperate and do not know what to do. With silence, they refuse, for example, to provide a specification or to propose their own solution.

In turn, therapists display that they expect further explanations or elaborations by one or several continuer(s), but they do not produce a full turn. Since the patients stay silent and do not show any intention to continue their turn, therapists end the silence with different interventions: questions (see Spranz-Fogasy, 2020), interpretations (see Vehviläinen, 2003), challenges (see Voutilainen et al., 2018) and formulations (see Weiste and Peräkylä, 2013). All these interventions have in common that they stay on topic and either focus on a specific aspect or open up a new perspective for the patient. In any case, they demand further engagement with the topic. Especially with questions (see Steensig and Drew, 2008), but also with other types of interventions, therapists make an answer conditionally relevant and require further reflection on the topic from P. Thereby, they characterize the previous silence often as disaligning and might give an implicit hint in which direction the patients should develop their self-reflection. Through interpretations and challenges, the therapists introduce a new aspect to the existing topic while they use questions and formulations to focus on a previous aspect of the narrative.

## 5 Discussion

The fact that therapists and patients construct silence together in all the cases presented in this article is essential. Although in some contexts (cf. 4.2), the patient’s silence might indeed be construed as defense or even resistance, and the therapist also indicates that s/he sees it as resistance, there are other contexts in which silence is certainly not resistance. In such contexts (cf. 4.1, 4.3, and 4.4), the patient’s silence could be interpreted, from a more relational perspective, as an action directed at the therapist as a request for his/her participation. When referring to silence as resistance, it can only be a shared resistance concerning the interactional demands. However, in the relational interpretation of silence, the patient, on the one hand, implicitly shows that s/he is asking for help from the therapist. The therapist, on the other hand, by remaining silent, ascribes to the patient the competence to proceed alone. These findings are in line with the so-called ‘two-person psychology,’ that is, the relational view of psychoanalytic therapy (see Mitchel, 1997, especially the introduction; Schwartz, 2012).

The results of the present study confirm the findings of Chatwin et al. (2018), who notice some discrepancies between the projections and the understanding of the interlocutors. Knol et al.’s (2020) study is the one that corresponds closest to mine. However, I can only confirm parts of their findings. According to my results, it is certainly true “that silence […] can become shaped as an intra-topic silence” (Knol et al., 2020). Although their investigation is too formal-structural and therefore not participant-adequate, calling it an “intra-topic silence” does not exactly describe what the pause is about, namely, disagreements in turn-taking and that the pause is not necessarily understood by the patients as intra-topic silence at first. Patients might even display a topic closure. Moreover, I have shown that the following four therapeutic actions address the topic further: questions, interpretations, challenges, and formulation. While especially questions and formulations are used to focus on a specific aspect of the topic, interpretations and challenges open up a new perspective. The former more strongly indicate that the topic itself has been incompletely dealt with regarding psychodynamic reflection and experienced emotions, whereas the latter puts the focus on therapy-relevant aspects.

Furthermore, in my data, I had no case of silence that was “explicitly characterized as an activity in itself that is relevant for the therapy in process” (Knol et al., 2020), that is, where silence is addressed in the form of a metacommunicative description. König (1995, p. 78) points out that it might help if the therapist asks the patient to verbalize the thoughts s/he had during the silence. In this way, the therapist implicitly repeats the permission to say everything, even if it is, for example, associated with shame. With such an intervention, the patient would be taken out of his/her ‘comfort zone’, and a possible resistance would be addressed more directly. One reason why this does not appear in my data could be that the therapists are still in training and are, therefore, more reticent to confront the patient or to address possible resistance, as they have little experience in this respect. Another reason could be that the treatments examined are psychodynamic psychotherapies, in which interventions encouraging free association are not such an integral part as this is the case in psychoanalysis.

In general, this article can contribute to increasing therapists’ awareness of silence and the various forms of silence that may occur in psychotherapy. The analysis reveals that in the case of silence, it is not only the absence of speech that must be considered but also the previous context and embodied behavior (including gaze). It is apparent that the silence investigated is often (at least implicitly) treated as disaligning—both by patients and therapists. However, this does not necessarily imply resistance. Instead, patients frequently request assistance indirectly when remaining silent. Furthermore, silence is no unilateral form of, for example, disalignment but is produced jointly by both participants in the conversation and can, therefore, be described as a shared interactional mean. Moreover, the study may help psychotherapists differentiate between four central therapeutic interventions and understand their functions, providing strategies on how to apply them effectively in the context of silence.

## Data Availability

The datasets presented in this article are not readily available because only project members are allowed to work with the data. For further enquiries please contact the author at fenner@ids-mannheim.de.

## Footnotes

^1^Psychodynamic psychotherapy emerges from psychoanalytic psychotherapy (see Abrahams and Rohleder, 2021).

^2^Of course, the therapist can also use silence, for example, as a ‘technical tool’ (cf. Thomä and Kächele, 2006, p. 264).

^3^For forms of productive silence, see, for example, Levitt (2002).

^4^In this article, I adopt a technical concept of silence in the sense of not speaking when the right to speak has been assigned.

^5^By using the term ‘narrative’, I refer not only to the telling of an event in the past (so-called storytelling, see Mandelbaum, 2013) but also to the telling of thoughts, wishes, feelings, and so on, no matter whether they are solicited by therapists’ questions or regularly volunteered by patients (cf. Bercelli et al., 2008).

^6^I did not include cases in which therapists and patients simultaneously end the silence.

^7^These four cases are too few to be able to say anything about what a change of topic means in such a sequential environment.

^8^In all examples, I will abbreviate patient and therapist as P and T, respectively.

^9^In order to keep the transcripts in the article readable and more concise, multimodal transcription was only done in parts relevant to the analysis. Note that I use -g for gaze and all other notations for other embodied conducts.

^10^Since T cannot be seen from the front on the video, clear head movements determine the direction of gaze.

^11^The video data are not primary but secondary data, and due to the resolution, I could only roughly recognize eye changes.
