# Supplementary material for: Silence after narratives by patients in psychodynamic psychotherapy: a conversation analytic study
Source: Front Psychol. 2024 Nov 28;15:1397523. doi: 10.3389/fpsyg.2024.1397523 (PMC11638706; doi:10.3389/fpsyg.2024.1397523)
Supplement: Supplementary file 1 [file Table_1.docx]

**Appendix 1**

Selected glossary of GAT 2 transcription conventions (cf. Selting et al., 2011, p. 37–39)

Sequential structure

[ ]
[ ] overlap and simultaneous talk
= fast, immediate continuation with a new turn or segment (latching)

Other segmental conventions

: lengthening, by about 0.2-0.5 sec.
:: lengthening, by about 0.5-0.8 sec.
ʔ cut-off by glottal closure

In- and outbreaths

°h / h° in-/outbreaths of appr. 0.2-0.5 sec. duration
°hh / hh° in-/outbreaths of app. 0.5-0.8 sec. duration

Pauses

(.) micro pause, estimated, up to 0.2 sec. duration appr.
(-) short estimated pause of appr. 0.2-0.5 sec. duration
(--) imtermediary estimated pause of appr. 0.5-0.8 sec. duration
(0.5) / (2.0) measured pause of appr. 0.5 / 2.0 sec. duration (to tenth of a second)

Laughter

ha ha / hi hi syllabic laughter
<<laughing> > laughter particles accompanying speech with indication of scope
<<:-)> > smile voice (with indication of scope)

Continuers / Response particles

hm, yes monosyllabic tokens
hm_hm bi-syllabic tokens

Final pitch movements of intonation phrases

? rising to high
, rising to mid
– level
; falling to mid
. falling to low

Accentuation

SYLlable focus accent
sYllable secondary accent

Loudness and tempo changes, with scope

<<p> > piano, soft
<<all> > allegro, fast

Other conventions

((name partner)) name omitted due to anonymization
(xxx) unintelligible syllable
(may I) assumed wording
(would have/has) possible alternatives
and_uh clitizations within units
uh, uhm, etc. hesitation markers

**Appendix 2**

Selected glossary of Mondada’s (2018, p. 106) multimodal transcription conventions

* * / + + description of embodied movements are delimited between two identical symbols (one symbol per participant’s line of action) and are synchronized with corresponding stretches of talk/lapses of time

*--> the action described continues across subsequent lines
-->* until the same symbol is reached
>> the action described begins before the extract’s beginning
-->> the action described continues after the extract’s end
...... preparation
------ full extension of the movement is reached and maintained
